# Identifying and Predicting Cognitive Decline Using Multi-Modal Sensor Data and Machine Learning Approach

**DOI:** 10.21203/rs.3.rs-6735622/v1

**Published:** 2025-06-18

**Authors:** Aparna Joshi, Jun Ha Chang, Guillermo Basulto-Elias, Shauna Hallmark, Matthew Rizzo, Anuj Sharma

**Affiliations:** Iowa State University; University of Nebraska Medical Center; Iowa State University; Iowa State University; University of Nebraska Medical Center; Iowa State University

**Keywords:** Mild Cognitive Impairment, Alzheimer’s, Naturalistic Driving, Digital Biomarker, Machine Learning, Leave-One-Subject-Out Approach

## Abstract

Alzheimer’s Disease (AD) remains a critical global health challenge, with its prevalence expected to rise dramatically by 2050, leading to substantial financial and emotional burdens. Mild Cognitive Impairment (MCI), the prodromal stage of AD, presents a crucial opportunity for early intervention, yet its diagnosis remains difficult due to the overlap with normal aging. Traditional diagnostic methods, such as neuroimaging and cerebrospinal fluid analysis, are costly and invasive, highlighting the need for alternative, scalable, and non-invasive biomarkers. This study explores the potential of naturalistic driving behavior as a digital biomarker for detecting cognitive decline in individuals at risk for AD and MCI. A total of 118 participants (8 with AD, 65 with MCI, and 45 cognitively healthy individuals) were included in this study. At baseline year, we measured their demographics, cognitive status administrated by dementia experts, 3 consecutive months of naturalistic driving performance and driving life-space from participants’ own vehicle and sleep data via wrist-worn actigraphy, integrated into multi-modal data to feed to XGBoost-based framework. After 1-year follow, their cognitive status was assessed. We implemented a two-phase validation framework: first, classification model using Leave-One-Subject-Out Cross-Validation (LOSO-CV) to classify baseline cognitive status, and then, conducting a prediction model with to assess the model’s ability to predict 1-year follow-up cognitive status. Our results demonstrate that the multi-modal classifier achieved strong classification performance (accuracy = 68.64%; precision = 73.97%; F1-score=74.48%), with the highest recall (76.39%) from a model incorporating demographics and driving features, and prediction performance (accuracy = 70.48%; precision = 71.88%; F1-score = 74.80%, recall = 77.97%). Key predictive features included sex, mean awakening duration, age, average acceleration, and sleep efficiency, underscoring the relevance of driving behavior and sleep characteristics in cognitive assessment. By leveraging everyday activities such as driving, this framework provides a novel, non-invasive approach for identifying individuals at risk for cognitive decline. Furthermore, its ability to predict future disease progression establishes a forward-looking paradigm for early detection and monitoring. Beyond cognitive impairment, this methodology offers a scalable and generalizable framework for disease prediction, with potential applications in detecting and monitoring other neurodegenerative and chronic conditions.

## Introduction

Alzheimer’s Disease (AD) is the only leading causes of death in the United States that cannot be prevented, slowed, or cured^1^. The World Alzheimer Report 2024 predicts that by 2050, the global number of individuals living with AD will rise to 139 million, with associated costs reaching 2.8 trillion dollars by 2030 in the US, imposing financial and emotional strain on family members and society^2^. Mild cognitive impairment (MCI), the prodromal stage of AD, involves more pronounced cognitive decline than normal aging, while preserving functional abilities, highlighting the need for early intervention^3^. MCI is not a definitive diagnosis of AD, but it plays a crucial role in predicting the onset of AD, with a conversion rate of 10~15% to AD^4,5^. Currently, AD pathology is primarily identified in research and clinical settings using imaging (e.g., amyloid positron emission tomography [PET]) or cerebrospinal fluid (CSF) biomarkers^6^ However, these methods are often limited by high cost, invasiveness, and logistical challenges. Bloodbased biomarkers (e.g., p-tau217) are emerging as promising tools with diagnostic performance approaching that of CSF assays. While recent criteria recognize their potential for diagnosis, they are still undergoing validation and have not yet been fully integrated into clinical practice^6^.

As the need for accessible, low-burden, and low-cost biomarker increases, digital biomarkers based on data from everyday technologies and activities has emerged as promising indicators of neurodegenerative disorders and overall brain health. Among them, naturalistic driving behavior has gained increasing interest for its potential to detect subtle cognitive and functional changes that are associated with preclinical AD and MCI^7,8^. As driving is a complex task that demands the integration of multiple cognitive domains, such as attention, memory, executive function, and visuospatial skills, within a dynamic environment, making it a practical, non-invasive, and sensitive marker of early cognitive decline^9^. Neurodegenerative diseases, including AD, have been shown to impair driving abilities and higher crash risk and more frequent unsafe driving behaviors^10^. Furthermore, specific driving patterns, such as speed and braking behavior, have been demonstrated to serve as predictors of driving safety in early AD^11^. Unlike road tests or simulators, naturalistic driving studies using in-vehicle sensors to capture behaviors in real-world condition, enhancing validity while reducing observer bias and test anxiety and reflecting progression of subtle cognitive decline^12–16^.

Machine learning (ML) techniques have been increasingly applied to use driving data for cognitive classification. Random Forest classifiers using demographic and driving features have shown strong performance in distinguishing MCI or preclinical AD, with F1 scores exceeding 0.85^8,17^. However, most existing studies face three key limitations. First, they primarily focus on cross-sectional classification, rather than predicting future cognitive decline, limiting clinical utility for early detection and intervention. Second, most models rely on driving data, rather than multi-model integration. For example, combining behavioral and physiological signals such as wearable-based sleep metrics could have the potential to improve discriminatory power. Third, many studies may suffer from data leakage, where individual-specific patterns inadvertently affect both training and testing sets, results in overfitting and poor generalizability to new individuals^18–20^.

Sleep, in particular, represents a critical yet underused dimension. Sleep disturbances are prevalent in individuals with AD, and have been linked to cognitive decline and amyloid accumulation^21,22^. Recent study has demonstrated associations between non-rapid eye movement (NREM) sleep features and cognitive performance in individuals with mild to moderate AD^23^. Real-world actigraphy-based sleep measures have shown utility in discriminating cognitive impairment, and it may represent a predictive marker^24^.

In the present study, we aimed to address these gaps by developing a ML model that integrates multi-modal real-world data, including naturalistic driving behavior and actigraphy-based sleep data, to both identify and predict cognitive decline in older adults. In Phase 1, we developed and validated an XGBoost classifier using a nested cross-validation (CV) approach with Leave-One-Subject-Out (LOSO-CV) to identify driver’s baseline cognitive status, minimizing the risk of data leakage and improving generalizability. In Phase 2, we applied the same trained model to predict driver’s cognitive status after one year, enabling temporal validation. This two-phase framework allows us to assess both diagnostic and prognostic utility of real-world behavioral and physiological data streams for cognitive impairment. We hypothesized that integration of driving and sleep data would enable to distinguish and predict drivers with cognitive decline from those without cognitive decline, both cross-sectionally and prospectively.

## Results

### Participant characteristics

To provide a comprehensive understanding of the study population, we examined key demographic and driving characteristics of the participants for each group (Table 1). The study included a total of 118 participants, with 76 classified as cognitively impaired (MCI/Alzheimer’s) and 42 as healthy aging. Due to the small number of participants classified as dementia (N = 8), this group combined with those classified as MCI (N = 64) to form a single unified cognitive impairment group (N = 72). The remaining participants (N = 46) were classified as the healthy aging. This binary classification (cognitive impairment vs. healthy aging) was used for all subsequent modeling to enhance statistical power while preserving the clinical continuum of cognitive decline. The average age of participants was 76.22 ± 6.03 years, with an insignificant difference between the healthy aging and cognitively impaired groups (p = 0.135). The proportion of female participants was higher in the healthy aging group (69.57%, *n* = 32) compared to the cognitively impaired group (40.28%, *n* = 29, p < 0.001). The overall mean years of education was 15.93 ± 2.54, with no significant difference between groups (p = 0.811).

Driving behavior metrics revealed notable differences. Participants in the healthy aging group had a higher total mile driven (140.79 ± 176.54) compared to the cognitively impaired group (121.77 ± 139.53, p = 0.027). Additionally, the average miles per trip were higher for the healthy aging group (6.29 ± 6.57) than for the cognitively impaired group (5.28 ± 3.87, p < 0.001). However, there were no significant differences in total number of trips or total trip minutes.

Regarding time-of-day driving patterns, cognitively impaired participants took slightly more nighttime trips than the healthy aging group (0.51 ± 1.44 vs. 0.37 ± 0.88, p = 0.024), though the percentage of trips taken at night was not significantly different. The number of trips taken during peak PM hours was significantly higher in the cognitively impaired group (p = 0.023).

Sleep-related metrics also showed significant differences, with the cognitively impaired group exhibiting lower mean sleep efficiency (0.82 ± 0.10, p = 0.002) and longer mean wake after sleep onset (83.03 ± 54.58 minutes) compared to the healthy aging group (73.89 ± 40.59 minutes, p < 0.001).

### Model Performance

#### Phase 1: Classification of Baseline Cognitive Status (Year *t*):

The accuracy, precision, recall, and F1-scores for each model are presented in Table 2. Single-feature models yielded moderate F1-scores: 62.86% (driving), 67.15% (demographics), and 68.12% (sleep). Combining driving features with demographic features, the model achieved an F1-score of 73.83% (model 4) and F1-score of 69.50% (model 5), respectively, resulting in an improved overall model performance. Finally, the model 6 integrating demographics, driving performance, driving life-space, and sleep data achieved the highest accuracy (68.64%), precision (73.97%), and F1-score (74.48%).

**Figure 1:**
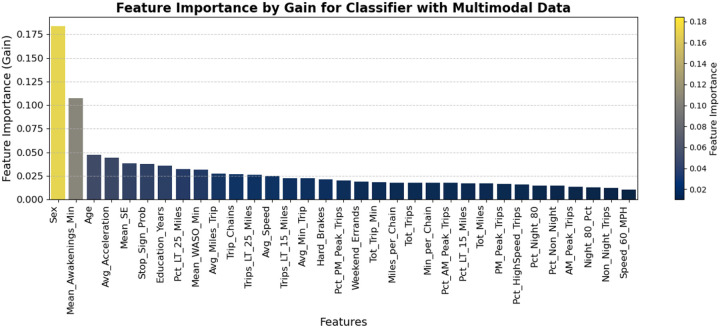
Feature Importance Ranking using Gain Metric

The five important features in the final model (model 6) that captured demographic, driving performance, driving life-space, and sleep are sex, average duration of awakening (mean_awakenings_min), age, average acceleration of trips (Avg_Acceleration), and average sleep efficiency (Mean_SE) (Figure 1).

#### Phase 2: Prediction of Cognitive Status at 1-Year Follow-Up (Year *t* + 1):

Thirteen participants in CI group were excluded due to missing cognitive status at year *t* + 1. All features remained unchanged from year *t*, with the outcome redefined as cognitive status at follow-up (1 = CI; 0 = HA), consistent with Phase 1 encoding. As shown in Table 3, the model demonstrated stable and slightly improved predictive performance for year *t+1*. Accuracy increased from 68.64% to 70.48%, and recall from 75.00% to 77.97%, while precision slightly decreased from 73.97% to 71.88%, reflecting common trade-off between improved sensitivity and a moderate increase in false positives. F1-score rose marginally from 74.48% to 74.80%.

## Discussion

This study presents a robust two-phase validation framework to evaluate the performance and predictive capacity of an XGBoost-based classification model for predicting Mild Cognitive Impairment (MCI) and AD. By integrating multi-modal data, including demographics, driving performance metrics, driving life-space variables, and sleep patterns, the model captures a comprehensive view of participants’ cognitive and functional status.

In Phase 1, a nested cross-validation approach with Leave-One-Subject-Out (LOSO-CV) robust model evaluation and provided an unbiased estimate of generalizability to unseen data. This methodological rigor enhances the reliability and clinical relevance of the findings. Our results demonstrate that the multi-modal model consistently outperforms single-variable models, achieving the highest accuracy (68.64%), precision (73.97%), and F1-score (74.48%). Interestingly, a model using only demographics and driving features achieved the highest recall (76.39%), suggesting their strong association with cognitive status.

Temporal validation in Phase 2 further demonstrated the model’s utility in forecasting cognitive status one year ahead, with improved accuracy (70.48%) and recall (77.97%). Key predictive features included sex, mean awakening duration, age, average acceleration, and sleep efficiency, highlighting the importance of both behavioral and physiological data in cognitive assessment. These findings demonstrate the potential of combining machine learning with real-world multi-modal data to advance the precision of cognitive health assessments. The ability to identify and predict cognitive decline using passive, non-invasive, and routinely collected data (i.e., driving, sleep) offers a scalable and accessible approach to early detection of Alzheimer’s disease and related dementia (ADRD). This particularly relevant in community settings where traditional biomarkers (e.g., CSF, PET imaging) are often unavailable. Moreover, high recall in predictive performance suggests that such tolls may be especially useful for screening purposes even without in-person clinic visits, identifying individuals who require further diagnostic evaluation.

This study has several notable strengths. First, it leverages naturalistic data from real-world driving patterns, providing valuable insights into participants’ everyday behaviors and offering a more accurate reflection of their daily lives. The use of multi-modal data, including driving patterns, demographics, and sleep behaviors, enhances the model’s predictive power by capturing a comprehensive view of cognitive and functional status. A key methodological strength is the application of nested LOSO-CV, which ensures unbiased evaluation and avoids data leakage, offering performance metrics that reflect the model’s generalizability to unseen data. Furthermore, the model’s ability to predict cognitive status one year ahead represents a significant advancement, pushing the field forward by enabling long-term forecasting of cognitive decline—a crucial step for early identification and intervention in clinical settings. Beyond cognitive impairment, this framework provides a scalable and generalizable methodology for disease prediction and progression modeling. By integrating multi-modal data and applying rigorous validation techniques, this approach can be adapted to detect and monitor other chronic conditions, such as neurodegenerative diseases, cardiovascular disorders, or metabolic syndromes. The ability to capture early behavioral and physiological changes through real-world data highlights the potential of this framework as a valuable tool for proactive healthcare and precision medicine.

The findings of this study should be interpreted with consideration of several inherent limitations that future research can address. First, the driving features used may not be comprehensive enough to optimize predictive performance. Incorporating more granular metrics—such as driving consistency, decision-making accuracy, and reaction time—could better capture subtle cognitive and motor changes preceding impairment. Additionally, expanding the sample size and including sociodemographic factors like race could improve model generalizability. Further, as all participants were from Nebraska and surrounding areas, the findings may not extend to other populations. Future research should develop separate models for MCI and Alzheimer’s disease, as well as for MCI-to-Alzheimer’s progression. Due to the small number of Alzheimer’s cases, MCI and Alzheimer’s were combined in this study; a larger, more balanced dataset could enhance predictive accuracy. Moreover, collecting multi-year data would allow models to predict disease status beyond a single year, strengthening their utility for early detection and intervention.

In conclusion, this study presents a promising framework for the objective and cost-effective prediction of cognitive decline in individuals at risk for ADRD. By leveraging multi-modal real-world data and a rigorous machine learning approach, this methodology offers a novel, scalable solution for early detection and intervention. Beyond ADRD, this framework can be adapted to monitor other neurodegenerative conditions, providing valuable insights for proactive healthcare and personalized treatment strategies. These findings contribute to advancing data-driven approaches for mitigating the impact of cognitive decline in aging populations.

## Methods

### Recruited participants

Legally licensed drivers, aged between 65 and 90, were recruited from the Omaha, Nebraska area between March 2021 and June 2024. Participants were recruited from the community through flyers, local news outlets, and presentations at senior organizations. The inclusion criteria included: 1) being aged between 65 and 90 years and living independently in Nebraska or the surrounding areas like Iowa; 2) holding a valid driver’s license and having at least 5 years of active driving experience; 3) being able to communicate in English; and 4) being capable of providing informed consent. All drivers chosen for this study met the Nebraska state driving license requirements, including a visual acuity of 20/40 or better (corrected or uncorrected). Any individuals with confounding medical conditions (e.g., pulmonary disease requiring chronic medication) or medications (e.g., stimulants) were excluded. Drivers consented to participate following institutional guidelines (University of Nebraska Medical Center [UNMC] IRB 522–20-FB).

### Data Sources

#### Driving Data:

A custom-built in-vehicle sensor system, referred to as the “Black Box”, was installed in each participant’s personal vehicles to continuously recorded naturalistic driving behavior over three months at baseline year. The system was automatically activated at every on-ignition and deactivated at off-ignition, with each ignition cycle defined as a discrete “drive.” The Black Boxes collected multi-modal data streams, including video (both forward roadway and vehicle cabin), Global Positioning System (GPS), Inertial Measurement Unit (IMU), and On-board Diagnostic (OBD) data, such as throttle position, vehicle speed, and engine revolutions per minute (RPM). To characterize driving behavior within the real-world road environment, GPS data were mapped to Geospatial Information System (GIS) roadway databases, including U.S. Census TIGER files, Omaha and Iowa shapefiles, Highway Performance Monitoring System (HPMS), and OpenStreetMap (OSM) shapefiles. These GIS databases provided the speed limit data and different roadways type information, enabling the integration of driving behavior with environmental road features.

#### Cognitive Data:

Participants were classified into three groups: healthy aging (HA), MCI, or dementia, based on the 2018 National Institute on Aging-Alzheimer’s Association (NIA-AA) research framework for cognitive continuum staging^6,25^.

Each participant underwent a comprehensive neurological and neuropsychological assessments conducted by two dementia-specialist clinicians. Assessments adhered to the National Alzheimer’s Coordinating Center (NACC) Unified Data Set (UDS) version which includes a battery of cognitive measures^26^. Functional status was evaluated using the Functional Activities Questionnaires (FAQ)^27^ and Clinical Dementia Rating Scale (CDR Dementia Staging Instrument)^28^. Participants were categorized as CU if they showed no evidence of cognitive deficits or functional impairment compared to their age-, sex- and education-matching comparisons. Participants with cognitive decline but without substantial functional impairments were classified as having MCI. Those who showed both cognitive and functional decline were classified as dementia. Cognitive status was assessed at baseline year and 1-year follow-up with the identical procedure.

#### Demographics and Sleep Data:

All participants completed a standardized self-reported demographic questionnaire covering age, sex, race/ethnicity, and years of education at baseline year. As part of the socioeconomic survey, they also provided information on their home addresses, workplaces, and regular destinations, including locations for errands, social activities, and medical appointments.

Sleep was objectively monitored using the ActiGraph GT9X triaxial accelerometer (ActiGraph LLC), which participants wore on their non-dominant wrist at a 30Hz sampling rate for three-month concurrently with driving data [33]. The device was removed only for water-related activities or charging. In addition, participants completed daily sleep logs noting in-bed and out-of-bed times, with assistance from study partners when needed. Raw actigraphy data were synched and processed using the CentrePoint platform, applying a validated sleep-wake detection algorithm^29^. Sleep metrics were calculated by integrating both self-reported sleep logs and device-measured sleep data. Key sleep variables included the average duration (in minutes) of all awakening episode, wake after sleep onset (WASO), calculated by summing the wake epochs between sleep onset and offset, and sleep efficiency (SE), defined as the ratio of total sleep time to the total time spent in bed.

### Data Preparation and Feature Development

In this section, we outline the steps taken to prepare and aggregate data for model building. First, we focused on developing relevant features to characterize driving behavior. Each ignition on-off instance was classified as a “drive” or “trip” made by the driver. Driving performance and driving life-space metrics were extracted by aggregating data on a weekly basis, allowing us to capture enough variation in driving behavior while maintaining a reasonable level of granularity for predictive modeling. Each weekly record for every participant was treated as an independent data point. With 1,500 data points (906 from cognitive impairment group and 594 from healthy aging group), weekly aggregation balanced the sparsity of monthly or participant-level data and the noise of daily data. This approach enabled the model to capture dynamic changes in behavior while preserving meaningful insights. To address the potential data leakage from aggregating weekly data, we implemented a **Leave-One-Subject-Out Cross-Validation** approach^30^. This ensures that data from the same participant is never present in both the training and testing sets, effectively eliminating any risk of leakage. Additionally, we applied **nested cross-validation**^31^ to independently optimize hyperparameters, ensuring that all model tuning was done on data that was not used in testing. This framework guarantees that the model’s performance is evaluated on truly unseen data, improving its ability to generalize to new drivers and mitigating the risk of overfitting. The methodology is further elaborated in **Model Development Section** below.

For driving life-space, features such as total miles driven, number of trips, trip distance, and time spent driving were calculated. Additionally, metrics related to the timing and frequency of trips, including trips during peak hours, day, night, and weekend errand trips^32^, were derived to reflect the driving context. For driving performance, we calculated metrics like the number of trips on high-speed roads, average trip acceleration, and average trip speed. To identify more specific driving behaviors, we also examined hard braking events (with deceleration rates of ≤ −0.35 g) and the probability of stopping at stop intersections. The latter was determined using computer vision techniques applied to video data, where we specifically identified stop intersections and then analyzed the driver’s stopping behavior at those intersections during the driving study period. Sleep data was processed by removing days when participants were not wearing the device, representing 5.3% of the total dataset.

The dataset included a total of 32 features, which encompassed not only driving performance, driving life-space metrics, and sleep variables, but also key demographic factors such as age, sex, and years of education. A detailed list of these multi-modal data features, along with their definitions, can be found in **Supplementary Table 1**.

### Model Selection

Extreme Gradient Boosting (XGBoost)^33,34^ was used for model development due to faster convergence, its computational efficiency, and strong regularization capabilities, which help mitigate overfitting. XGBoost was especially effective in our study because it compensates for class imbalance by giving more importance to underrepresented cases, based on the ratio of negative to positive samples. Compared to many other classification algorithms, XGBoost requires minimal feature engineering, eliminating the need for scaling or normalizing data, and its built-in handling of missing data^35^, making it highly practical for real-world behavior datasets like ours.

Our dataset showed class imbalance, with a higher proportion of Cognitively Impaired (coded as 1) compared to Healthy Aging (coded as 0) at baseline year. To address this, we calculated the scale_pos_weight parameter for the XGBoost model as the inverse class ratio (i.e., class 1 vs class 0), ensuring that the model to emphasize the minority class during training and improve performance under imbalance.

Missing data were handled based on the context and meaning of each variable. For example, missingness in the average distance and minimum duration per trip chain (Miles_per_Chain and Min_per_Chain, respectively) indicated no trip chains during the week and was thus recorded as 0 to preserve the interpretability of the data. Similarly, missing values for weekend errand trip were recorded as 0 to reflect the true trip count.

In contrast, missing values for Stop_Sign_Prob, representing the weekly proportion of stops at intersections, were retained as missing value (i.e., NaN). This is because missingness reflected the absence of encountering with stop intersections (65% of the dataset), and imputing a value of 0 would be misleading that driver failed to stop despite having the opportunity. XGBoost can handle missing values to process these cases without imputation.

Categorical variables such as sex and cognitive status were binary encoded to ensure compatibility with the XGBoost model. Sleep-related variables, such as Mean Sleep Efficiency, Mean Awakening Duration, and Mean Wake After Sleep Onset, contained about 10.8% of missingness, likely due to device malfunctions or incomplete collection. Rather than imputing missing values, we allowed XGBoost to handle these missing values naturally, leveraging its ability to incorporate missing data into its tree-based learning process without introducing artificial bias^35^.

### Nested Cross-Validation for Hyperparameter Tuning

This two-layer cross-validation (CV) approach is designed to optimize the model’s hyperparameters and assess its generalizability to unseen data^31^.

The **Inner CV loop** tuned the hyperparameters of the XGBoost model using RandomizedSearchCV^36^ with 10-fold stratified CV, ensuring that the training data is effectively utilized while preventing overfitting. The hyperparameters explored included the number of estimators, maximum depth, learning rate, regularization parameters (L1 and L2), and the row and column sampling rates. To mitigate class imbalance, scale_pos_weight was dynamically calculated for each training fold based on the ratio of the majority to minority class. This weight adjustment ensures that the model does not become biased toward the majority class, improving its ability to correctly classify the minority class. Additionally, the use of stratified CV in the inner loop (for hyperparameter tuning) ensures that each fold maintains the same class distribution as the entire training dataset. This further reduces the risk of overfitting to a particular class and improves the model’s ability to generalize. The final model selection is based on the F1-score, optimizing the balance between precision and recall.

The **Outer CV Loop** used Leave-One-Subject-Out Cross-Validation (LOSOCV) to evaluate generalization performance^30^. In each iteration, one participant was completely left out as the test set, while the remaining participants data were used for training and hyperparameter tuning within the inner loop. This approach ensured that each participant’s data served as a true unseen test data, providing a robust estimate of its real-world performance.

This approach ensured that each participant serves as an independent external test set exactly once, providing an unbiased assessment of the model’s ability to generalize across individuals. To improve computational efficiency, LOSOCV was parallelized using *joblib* in python^37^, distributing the workload across multiple processors. Once the data preprocessing and handling missing data was complete, we proceeded to train models using multi-modal data to capture a more comprehensive understanding of cognitive health.

### Multi-Modal Data for Building and Training Classification Models

Multi-modal data refers to the integration of information from different types of variables or data sources to train predictive models. In this study, we utilize three distinct modalities—demographics, driving life-space variables, driving performance, and sleep patterns—to capture a comprehensive understanding of the factors influencing the target variable. Each modality offers unique insights: demographics provide static background information, driving life-space reflects the extent and patterns of a participant’s driving activity, and driving performance captures behavioral tendencies and driving efficiency. Meanwhile, sleep data provides insights into physiological and lifestyle aspects. Six models were trained with XGBoost, each using different combinations of features to assess their impact on predicting cognitive health. The models were trained on the following feature sets: (1) Only demographics (e.g., age, sex, years of education), (2) Only driving performance features (e.g., trips on high-speed roads, average speed, hard braking events) and driving life-space features (e.g., total miles driven, trip duration, trip chains), (3) Only sleep features, (4) demographics, driving life-space and driving performance features, (5) Driving performance, driving life-space features and sleep patterns, and (6) all available features, including demographics, driving performance, driving life-space, and sleep patterns. By evaluating these different combinations, we can better understand how each modality contributes to the prediction of cognitive health.

### Model Evaluation Strategy

We used a two-phase evaluation strategy to address two objectives: (1) classification of cognitive status at baseline (year t), and (2) prediction of cognitive status at 1-year follow-up (year *t+1*). The same modeling framework was used across both phases, with the final model selected during Phase 1 applied directly to Phase 2.

#### Phase 1: Classification of Baseline Cognitive Status (Year *t*):

The primary objective in Phase 1 was to classify participants as cognitively impaired (CI) or healthy aging (HA) at baseline using multi-modal features of driving, sleep, and demographic data collected in year *t*.

**Figure 2:**
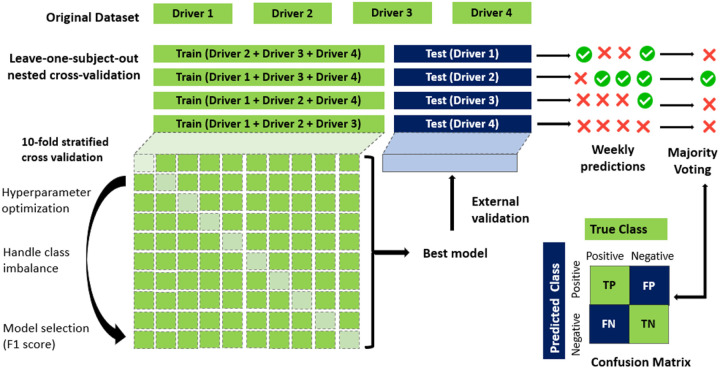
Proposed Framework for Nested Leave-One-Subject-Out Cross Validation

We computed multiple metrics, including accuracy, precision, recall, and F1-score, at the driver level rather than at the weekly observation level. This approach aligns with real-world clinical assessments, where cognitive status is assessed per individual. Given that each participant contributed multiple weekly records, we applied a **bagging-based aggregation approach** after each iteration of LOSO-CV. For the held-out participant, all weekly predictions were generated, and final classification was determined using majority voting (Equation 1):

$${\hat{\text{y}}}_{s}=\left\{\begin{array}{ll} 1, & \sum_{i=1}^{n_{s}}{\hat{\text{y}}}_{i}\geq \frac{n_{s}}{2}\\ 0, & \sum_{i=1}^{n_{s}}{\hat{\text{y}}}_{i}<\frac{n_{s}}{2} \end{array}\right.$$

where ${\hat{\text{y}}}_{s}$ is the subject-level classification (1 = CI, 0 = HA), ${\hat{\text{y}}}_{i}$ is the predicted weekly label, and n_s_ is the number of weekly data points available for driver s. This aggregation approach can mitigate short-term fluctuations and provides a more clinically stable cognitive classification. The proposed framework, which incorporates a nested LOSO-CV strategy along with a bagging-based aggregation approach, is illustrated in Figure 2.

Model selection was guided by the F1-score to balance precision and recall. Performance metrics were calculated based on aggregated subject-level predictions, and a confusion matrix was generated to summarize classification outcomes. The best-performing model from Phase 1 was retained without retraining and applied directly in Phase 2.

#### Phase 2: Prediction of Cognitive Status at 1-Year Follow-Up (Year *t* + 1):

**Figure 3:**
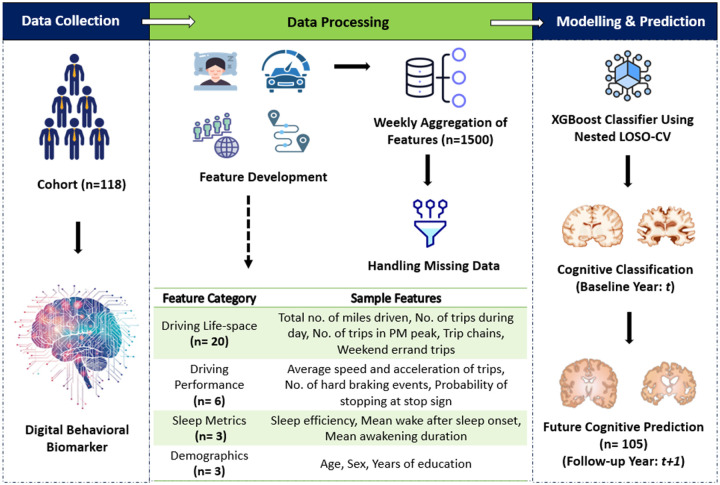
Methodology Pipeline for Developing Digital Biomarker

In Phase 2, we evaluated the generalizability and predictive capability of the final XGBoost classifier developed in Phase 1 to predict cognitive status one year later, using only baseline data (year *t*) as input. No additional model tuning or retraining was performed. Participants missing cognitive status at year *t + 1* were excluded from this phase. All feature inputs remained unchanged from year t, while the outcome variable was defined as cognitive status at year *t + 1* (1 = CI, 0 = HA). Figure 3 illustrates the methodological pipeline used to develop the digital biomarker in this study.

In sum, our findings demonstrate that passively collected driving and sleep data, when combined with demographic factors, can accurately predict future cognitive status in older adults. This approach offers a scalable, non-invasive, and ecologically valid method for early risk stratification. While promising, further research is needed to validate the approach. Although the sample size was modest and AD cases limited, our results provide a proof-of-concept for larger, more diverse studies integrating real-world data for early cognitive risk stratification. Future efforts will focus on external validation, implementation in clinical workflows, and real-time monitoring to support early intervention and personalized care^38^.

## Supplementary Material

Supplementary information

This study contains supplementary material.

Supplementary Files

This is a list of supplementary files associated with this preprint. Click to download.

- SupplementarydocumentAJ.docx

## Figures and Tables

**Table 1:** Descriptive analysis of demographics and weekly aggregated driving and sleep features

Feature Name	Overall (n=118)	Healthy Aging (n= 46)	Cognitively Impaired (n=72)	p-value (chi-square/t-test)
Age	76.22 ± 6.03	75.87 ± 6.29	76.21 ± 5.52	0.135
Sex (Female), n (%)	61 (51.69%)	32 (69.57%)	29 (40.28%)	**< 0.003** *
Education (years)	15.93 ± 2.54	15.88 ± 2.71	16.00 ± 2.28	0.811
Total Miles Driven	129.30 ± 155.47	140.79 ± 176.54	121.77 ± 139.53	**0.027** *
Total no. of Trips	21.80 ± 12.49	21.25 ± 11.55	22.15 ± 13.07	0.163
Average Miles per trip	5.68 ± 5.13	6.29 ± 6.57	5.28 ± 3.87	**< 0.001** *
Average Minute per trip	14.72 ± 22.41	14.18 ± 7.46	15.07 ± 28.19	0.370
Total trip minutes	314.07 ± 275.72	303.88 ± 233.95	320.75 ± 299.91	0.223
No. trips during day	21.34 ± 12.07	20.88 ± 11.20	21.65 ± 12.61	0.221
% trips during day	98.23 ± 5.50	98.48 ± 5.14	98.06 ± 5.73	0.145
No. trips in AM peak	3.51 ± 3.47	3.37 ± 3.21	3.60 ± 3.63	0.189
% trips in AM peak	16.04 ± 14.90	15.56 ± 13.92	16.36 ± 15.51	0.295
No. trips at night	0.45 ± 1.25	0.37 ± 0.88	0.51 ± 1.44	**0.024** *
% trips at night	1.77 ± 5.50	1.52 ± 5.14	1.94 ± 5.73	0.145
No. trips in PM peak	3.71 ± 3.45	3.57 ± 3.13	3.81 ± 3.64	**0.023** *
% trips in PM peak	16.87 ± 15.08	16.92 ± 14.88	16.84 ± 15.22	0.922
No. trips < 15 miles of home	20.36 ± 11.65	19.71 ± 10.80	20.80 ± 12.17	0.069
% trip < 15 miles of home	93.83 ± 10.85	93.11 ± 11.60	94.30 ± 10.31	**0.044** *
No. trips < 25 miles of home	0.75 ± 1.45	0.73 ± 1.33	0.75 ± 1.52	0.790
% trip < 25 miles of home	3.27 ± 7.23	3.23 ± 6.61	3.29 ± 7.62	0.872
Trip chains	0.92 ± 1.77	0.68 ± 1.11	1.07 ± 2.08	**<0.001** *
Miles per chain	6.78 ± 11.99	6.74 ± 15.37	6.81 ± 9.23	0.954
Minutes per chain	20.65 ± 20.85	19.24 ± 23.51	21.55 ± 18.94	0.216
Weekend Errand Trips	2.38 ± 2.00	2.59 ± 1.96	2.24 ± 2.02	**0.034** *
No. trips on high-speed roads	0.03 ± 0.10	0.02 ± 0.10	0.03 ± 0.09	0.175
*%* trip on high-speed roads	19.68 ± 5.99	20.11 ± 5.89	19.4 ± 6.04	**0.019** *
Average acceleration	0.03 ± 0.10	0.02 ± 0.10	0.03 ± 0.09	**0.041** *
Average speed	19.77 ± 6.12	20.11 ± 5.88	19.54 ± 6.26	**0.025** *
No. hard braking events with deceleration rates <= −0.35 g	1.41 ± 1.63	1.42 ± 1.75	1.40 ± 1.55	**0.804**
Probability of stopping at stop sign	0.84 ± 0.31	0.88 ± 0.26	0.81 ± 0.33	**0.005** *
Mean Sleep Efficiency	0.82 ± 0.09	0.83 ± 0.09	0.82 ± 0.10	**0.002** *
Mean Awakening Duration	5.05 ± 2.88	4.38 ± 1.93	5.51 ± 3.32	**<0.001** *
Mean Wake After Sleep Onset	79.28 ± 49.51	73.89 ± 40.59	83.03 ± 54.58	**<0.001** *

Note:

* Results indicate statistical significance at an error rate of 5%. Values are presented as mean ± standard deviation unless otherwise noted.

**Table 2:** Model Metrics for XGBoost Classifiers

Covariates	Accuracy	Precision	Recall	F1 Score
Model 1: Only Demographics	61.86%	70.77%	63.89%	67.15%
Model 2: Only Driving Features	55.93%	64.71%	61.11%	62.86%
Model 3: Only Sleep Features	62.71%	71.21%	65.28%	68.12%
Model 4: Demographics + Driving	66.95%	71.43%	**76.39%**	73.83%
Model 5: Driving + Sleep	63.56%	71.01%	68.06%	69.50%
Model 6: Multi-Modal Data	**68.64%**	**73.97%**	75.00%	**74.48%**

**Table 3:** XGBoost Classification Results for Predicting Future Cognitive Status

Covariates	Accuracy	Precision	Recall	F1 Score
***t* year:** Multi-Modal Data	68.64%	**73.97%**	75.00%	74.48%
***t+1* year:** Multi-Modal Data	**70.48%**	71.88%	**77.97%**	**74.80%**

## Data Availability

Given the sensitive nature of the data collected in this study, participants were guaranteed that the raw data would remain confidential and would not be disclosed.
